# The effects of a protein enriched diet with lean red meat combined with a multi-modal exercise program on muscle and cognitive health and function in older adults: study protocol for a randomised controlled trial

**DOI:** 10.1186/s13063-015-0884-x

**Published:** 2015-08-08

**Authors:** Robin M. Daly, Jenny Gianoudis, Melissa Prosser, Dawson Kidgell, Kathryn A. Ellis, Stella O’Connell, Caryl A. Nowson

**Affiliations:** Centre for Physical Activity and Nutrition Research, Deakin University, Melbourne, Australia; Department of Rehabilitation, Nutrition and Sport, College of Science, Health and Engineering, La Trobe University, Melbourne, Australia; Academic Unit for Psychiatry of Old Age, Department of Psychiatry, The University of Melbourne, Melbourne, Australia

**Keywords:** Red meat, Progressive resistance training, Muscle, Cognition, Older adults, Randomised controlled trial, Study protocol

## Abstract

**Background:**

Age-related muscle wasting has been strongly implicated with falls and fractures in the elderly, but it has also been associated with cognitive decline and dementia. Progressive resistance training (PRT) and adequate dietary protein are recognised as important contributors to the maintenance of muscle health and function in older adults. However, both factors also have the potential to improve brain function and prevent cognitive decline via several pathways, including the regulation of various growth and neurotrophic factors [insulin-like growth factor-1 (IGF-1)]; brain-derived growth factor (BDNF)] and/or the modulation of systemic inflammation. The primary aim of this study is to investigate whether a modest increase in dietary protein achieved through the consumption of lean red meat three days per week, when combined with PRT, can enhance muscle mass, size and strength and cognitive function in community-dwelling older people.

**Methods/Design:**

The study design is a 48-week randomised controlled trial consisting of a 24-week intervention with a 24-week follow-up. Men and women (*n*=152) aged 65 years and over residing in the community will be randomly allocated to: 1) PRT and provided with 220 g (raw weight) of lean red meat to be cooked and divided into two 80 g servings on each of the three days that they complete their exercise session, or 2) control PRT in which participants will be provided with and advised to consume ≥1 serving (~1/2 cup) of rice and/or pasta or 1 medium potato on each of the three training days. The primary outcome measures will be muscle mass, size and strength and cognitive function. Secondary outcomes will include changes in: muscle function, neural health (corticospinal excitability and inhibition and voluntary activation), serum IGF-1 and BDNF, adipokines and inflammatory markers, fat mass and inter-/intra-muscular fat, blood pressure, lipids and health-related quality of life. All outcome measures will be assessed at baseline and 24 weeks, with the exception of cognitive function and the various neurobiological and inflammatory markers which will also be assessed at week 12.

**Discussion:**

The findings from this study will provide important new information on whether a modest increase in dietary protein achieved through the ingestion of lean red meat can enhance the effects of PRT on muscle mass, size and strength as well as cognitive function in community-dwelling older adults. If successful, the findings will form the basis for more precise exercise and nutrition guidelines for the management and prevention of age-related changes in muscle and neural health and cognitive function in the elderly.

**Trial registration:**

Australian New Zealand Clinical Trials Registry: ACTRN12613001153707. Date registered 16^th^ October, 2013.

## Background

Age-related loss in muscle mass, strength and function, which has been termed sarcopenia, has been linked to a range of common chronic diseases, including an increased risk for falls and fractures [1–3]. Although there is no single cause of sarcopenia, physical inactivity, inadequate nutrition and age-related reductions in circulating sex steroids, growth factors and vitamin D, and concomitant increases in pro-inflammatory cytokines such as interleukin-6 (IL-6), C-reactive protein (CRP) and tumor necrosis factor-alpha (TNF-α), have all been implicated [2, 4]. There is also strong evidence that dementia and related cognitive deficits in older people, particularly deficits in executive function such as the ability to concentrate, to attend selectively, multi-task and to plan and strategize, are associated with both risk factors for falls [e.g., postural instability, impaired gait, reduced ability to perform activities of daily living (ADL)] as well as future falls [5]. Other studies have also reported that low muscle mass and strength and muscle loss are associated with cognitive impairment [6–8] and brain atrophy [9]. Thus, identifying interventions that can optimize muscle mass, strength and function, and improve cognitive function are likely to provide the greatest benefits in terms of ensuring that older adults can live independently and relatively disease and disability free into old age.

Progressive resistance training (PRT) is promoted as one of the few lifestyle approaches that can significantly increase muscle strength, mass and size, but there remains considerable heterogeneity in the skeletal muscle responses to PRT in older adults [10, 11]. For muscle hypertrophy to occur there must be a net increase in muscle protein balance, that is, muscle protein synthesis (MPS) must exceed muscle protein breakdown (MPB). PRT can stimulate MPS above resting levels, but MPB also rises such that net protein balance remains negative in the fasted state [12]. While the provision of protein (amino acids) post-exercise can overcome this imbalance and result in a net positive protein balance, the anabolic sensitivity of skeletal muscle to protein appears to be blunted with ageing [12]. As a result, it has been suggested that older adults require a higher dose of protein to maximise MPS and promote muscle hypertrophy in response to PRT [12, 13]. Although the optimal dose of protein needed to enhance the anabolic response to PRT in older adults is not known, there is some evidence that a daily protein intake >1.2 to 1.5 g/kg body weight or 20–40 g of high quality protein ingested following PRT can promote MPS and muscle hypertrophy [13–16]. While most previous studies have focused on the effects of milk (whey and casein) and soy protein, red meat is another rich source of protein that contains complete and balanced proportions of all eight essential amino acids, and has been shown to promote MPS at rest and following PRT in older adults and the elderly [17–20]. For instance, it has been shown that ingestion of a 113 g (moderate) serving of beef (30 g protein) produced a 50 % increase in MPS [18], with no further gains following the ingestion of a larger 340 g serving (90 g protein) [20]. In a recent 4-month randomised controlled trial (RCT) in women aged 60 years and over, we found that ingestion of two ~80 g servings of cooked lean red meat on most days of the week enhanced the effects of PRT on muscle mass, strength and serum insulin-like growth factor-1 (IGF-1) and reduced inflammation [serum IL-6]; there were no adverse effects on blood pressure, lipids or kidney function [15]. Despite these positive findings, promoting daily consumption of red meat is not consistent with the current Australian Dietary Guidelines [21]. Thus further research is needed to determine whether less frequent consumption (e.g., an intake consistent with current dietary guidelines of no more than 455 g per week of cooked lean red meat) is equally effective when consumed in combination with exercise.

In addition to preserving muscle health in older people, recent attention has focused on identifying strategies to attenuate age-related cognitive declines as this has been identified as a key risk factor associated with falls and disability in the elderly [22], and precedes the development of dementia and Alzheimer’s disease [23]. Globally the prevalence of dementia is estimated to increase from around 35 million people in 2010 to around 115 million people by 2050, with many more people having some degree of cognitive impairment [24]. Currently there are no disease modifying pharmacological treatments to cure or delay the progression to dementia. However, regular exercise, including aerobic training, PRT or the combination, has been recognised as promising strategies that can induce a cascade of molecular and cellular processes which may improve brain function, structure and/or neural connectivity via several pathways, including the release of various growth and neurotrophic factors, and cognitive function, particularly measures of executive functioning and processing speed [25–29]. However, not all studies have reported beneficial effects of exercise on measures of cognition, which may relate to differences in the study populations, study duration, training dose (duration, intensity and/or frequency) and/or the dietary habits of individuals.

Previous research has shown that certain dietary patterns or nutrients can have a positive effect on neuronal plasticity and cognitive function among older adults [30–33]. Although the data from randomised controlled trials is limited, a number of reviews have highlighted that the findings from epidemiologic studies tend to support a protective role of omega-3 fatty acids, B-vitamins or antioxidants and the Mediterranean diet [30, 32, 33]. There is also emerging evidence that dietary protein and its constituent amino acids are associated with cognitive function in older adults [34–36]. In a population-based, prospective study (median follow-up 3.7 years) in 937 elderly aged 70–89 years who were cognitively normal at baseline, a higher dietary protein intake was associated with a 21 % reduced risk of mild cognitive impairment or dementia, independent of potential confounders [37]. While there are few intervention trials which have examined the effects of dietary protein or protein supplementation on cognition, a study in healthy young men demonstrated that a high-protein meat diet for 3 weeks improved cognitive function (reaction time for demanding tasks) [38]. Moreover, an Australian study reported that women habitually consuming less than the recommended intake of red meat were twofold more likely to experience depressive or anxiety disorders than those consuming the recommended amount [39]. Given that red meat contains other nutrients in addition to protein such as iron, zinc, several B vitamins, omega-3 fatty acids and possibly vitamin D which have all been linked to cognitive function, we hypothesise that combining a diet rich in red meat with PRT will be more effective for enhancing cognitive function and neural plasticity in older adults than PRT alone.

There are several lines of evidence to support our hypothesis. One of the proposed mechanisms by which exercise may improve cognition and neural plasticity as well as muscle health is via an increase in circulating IGF-1 and serum brain-derived neurotrophic factor (BDNF) and/or a reduction in systemic inflammation [40, 41]. It is well known that IGF-1 is central to muscle growth, but it has also been shown to have neuroprotective actions by promoting neuronal growth, survival and differentiation [40, 42]. IGF-1 has also been shown to augment BDNF concentrations, which is a neurotrophin that functions to support the growth and maintenance of neuronal circuits as we age [40]. Indeed, a number of human studies have reported a link between circulating BDNF concentrations and cognition [43, 44], and there is evidence that BDNF stimulates neurons to survive longer and branch and connect in new ways (synaptic plasticity) to promote memory and learning [40, 45] as well as executive function and processing speed [44, 46]. Finally, increased inflammation has also been linked to muscle loss and cognitive impairment, and associated with an increased risk of dementia [47, 48]. Building upon our previous 4-month randomised controlled trial in elderly women which showed that PRT combined with increased consumption of lean red meat on most days of the week resulted in a greater increase in serum IGF-1 and a reduction in circulating IL-6 levels compared to PRT alone, together with existing research which has shown that dietary protein and exercise can stimulate an increase in serum BDNF, we hypothesise that combining these approaches will be more effective at improving cognitive performance in older adults than exercise alone. In addition, by measuring changes in cortical plasticity using Transcranial Magnetic Stimulation (TMS), a painless and non-invasive technique that allows an assessment of neuroplasticity through cortical excitability and inhibition (eg. responsiveness of the entire neural pathway from the motor cortex to the muscle), this study will provide new data on the role of PRT combined with increased dietary protein on neural plasticity in the elderly.

The primary aim of this 24-week randomised controlled trial with a 24-week follow-up in community-dwelling older adults is to examine the effects of a multi-modal exercise program involving PRT combined with a protein-enriched diet achieved through lean red meat consumed three days per week (on training days) on skeletal muscle mass, size and strength, and cognitive function. The secondary aims of the study are to:

1. Evaluate the effects of the intervention on physical functioning, neural plasticity (corticospinal excitability and inhibition and voluntary activation), various growth and neurotrophic factors and systemic inflammatory markers associated with muscle and cognitive health, and health-related quality of life;
2. Explore whether any intervention related changes in cognitive function are modulated by body composition, neurotrophic markers, inflammatory cytokines, circulating growth factors, bioavailable zinc and cardiovascular risk factors;
3. Determine if the 24-week supervised and structured exercise and protein intervention can lead to long-term behavioural changes with regard to maintaining participation in exercise and/or consuming a diet rich in red meat in older adults.

## Methods

### Study design

This is a 48-week randomised controlled trial consisting of a 24-week intervention with a 24-week follow-up. Men and women aged 65 years and over will be randomly allocated to one of two groups: 1) PRT with two 80g servings of cooked lean red meat on each of the three days that they complete their exercise session (RT+Meat group), or 2) control PRT in which participants will be provided with and advised to consume ≥1 serving (~1/2 cup) of rice and/or pasta or 1 medium potato on each of the three training days [control resistance training (CRT)]. On the non-training days, the red meat group will be instructed to maintain their usual diet whilst those in the CRT group will be asked to continue to incorporate larger servings of breads, cereals, rice, pasta and vegetables (carbohydrates) and smaller amounts of protein foods (e.g. meat, fish and chicken). All participants will be asked to take a daily vitamin D supplement (1000 IU) and encouraged to maintain a healthy diet that includes the recommended amounts of fruits and vegetables and dietary calcium throughout the entire study. At the completion of the 24-week intervention, all participants will be encouraged to continue to undertake the exercise program (at their own expense) and those in the red meat group were provided with dietary advice to maintain a protein rich diet (at least 1.2 g/kg/day) including red meat which will be monitored at weeks 36 and 48. The trial is managed by the Centre for Physical Activity and Nutrition Research at Deakin University, Burwood, Melbourne, Australia and is funded by a nationally competitive grant from Meat and Livestock Australia. The study has been approved by the Deakin University Human Research Ethics Committee (HREC 2013–166), and is registered with the Australian and New Zealand Clinical Trials Registry (ACTRN12613001153707).

### Participants

At least 152 community-dwelling men and women aged 65 years and over will be invited to participate in this study.

#### Recruitment

The intervention will be implemented across a number of local community-based health and fitness centres within Melbourne and surrounding areas in Victoria, Australia. Recruitment and testing of participants will be staggered and split into two cohorts over 2-years, each starting at the same time of the year, to facilitate the management and implementation of the trial. A variety of recruitment strategies will be employed to attract participants, including information talks at community centres, local media campaigns including newspaper and magazine advertisements, flyers placed on relevant notice boards and word of mouth. All participants that express an interest in being involved in the study will undergo screening to determine their eligibility to participate in the trial based on the criteria outlined below.

#### Screening and eligibility

Screening will consist of a four-step process. First, all participants will be screened over the telephone and deemed ineligible based on the following criteria: 1) aged <65 years; 2) current or prior participation in resistance exercise (>1 day per week) and/or moderate-intensity physical activity ≥150 minutes per week in the past 3 months; 3) acute or terminal illness, including current (treated or untreated) cancer or surgery/cessation of chemotherapy/radiotherapy less than 12-months ago; 4) history of a recent low trauma fracture with functional impairment that would limit participation in the exercise program; 5) body mass index (BMI) >40; 6) use of insulin to treat diabetes; 7) chronic liver disease, coeliac or inflammatory bowel disease, including ulcerative colitis or Crohn’s disease; 8) recent use of oral corticosteroids (past 6 months); or 9) inability to commit to the study and its requirements. Participants will not be excluded if they are currently taking vitamin D supplements, but will be asked to take the vitamin D that is provided to them as part of the study so that we are able to monitor compliance. Second, eligible participants will then be asked to complete the Short Portable Mental Status Questionnaire (SPMSQ) and the Geriatric Depression Scale (GDS) to evaluate whether they are cognitively healthy and have no signs of depression. Those with a score of greater than two on the SPMSQ and/or a score of greater than six on the GDS will be excluded from the study. For these participants, a letter will be sent to their doctor and they will be encouraged to make an appointment with them to discuss this is in further detail. Participants that remain eligible will then be required to obtain approval from their local doctor to clear them of any contraindicated medical conditions to exercise, based upon American College of Sports Medicine (ACSM) guidelines, to participate in the program. Finally, participants will be asked to provide a fasted, morning blood sample to confirm that their estimated Glomerular Filtration Rate (eGFR) is >45 mL/min/1.73m^2^. Written informed consent will be obtained from all participants prior to commencing the program.

#### Randomisation and blinding

**Fig. 1:**
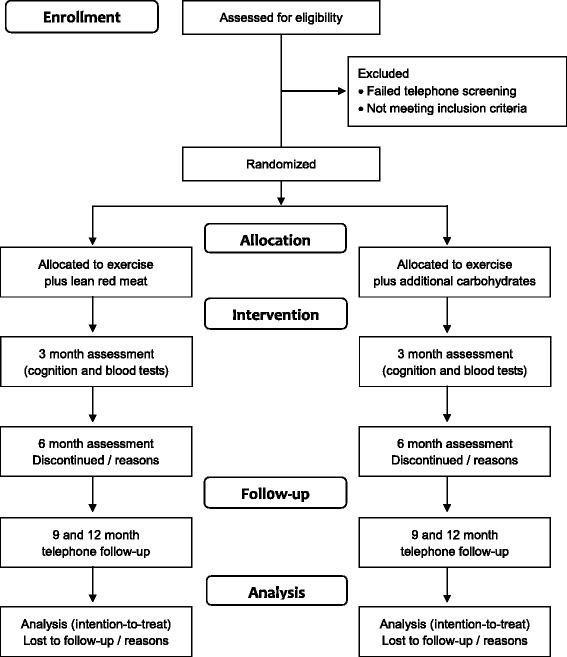
Flow diagram of the progress from screening to the final follow-up assessment

Randomisation will be at the level of the individual participant in blocks of four, stratified by gender, using a computer-generated random number sequence by an independent researcher. Neither the participants nor research staff involved in the testing will be blinded to the allocation, but those analysing the data will be blinded. A flow diagram of the study protocol is outlined in Fig. 1.

### Intervention

#### Exercise program

All participants involved in the study will be prescribed an individually tailored and supervised aerobic, PRT and challenging balance and mobility program. All training will be conducted at local community health and fitness centres and participants will be asked to train on three non-consecutive days per week for 24 weeks. Each training session will last approximately 60–75 minutes, and include: a warm-up and cool-down, 15–20 minutes of moderate intensity [5 to 8 (‘somewhat hard’ to ‘hard’) on the 10 point Borg Rating of Perceived Exertion (RPE) scale] aerobic training (e.g. cycling, treadmill walking, stepping), at least two challenging balance/mobility and postural exercises, and 30–40 minutes of moderate to high intensity PRT. All exercise sessions will be supervised by qualified exercise trainers and will be conducted in small groups where possible (approximately 6–8 per group), but each participant’s program will be individualised. For the first two weeks, participants will be asked to complete three sets of 12–15 repetitions for each resistance exercise using relatively moderate resistance to familiarize themselves with the equipment (free and machine weights), the training protocol, and the correct execution of the exercises. Thereafter, participants will be asked to complete three sets of 8–12 repetitions for at least eight different upper and lower body exercises at an intensity corresponding to 5 to 8 (‘somewhat hard’ to ‘hard’) on the 10 point Borg RPE scale. Some examples of the exercises that will be used throughout the program include: squats, lunges, box step-ups, leg extensions, standing leg curls, hip abductions, calf raises, shoulder press, upright row, bicep curls, wall push-ups, and triceps kickbacks/pushdowns. Where possible, all exercise sessions will be conducted prior to lunch or dinner so that the participants can consume their prescribed meal type as soon as possible following the training. If this is not possible, participants will be instructed to consume one serve of red meat before training or both serves after a training session.

#### Lean red meat

Participants allocated to the red meat group will be supplied with ~220 g (raw weight) of lean red meat to be consumed on each of the three training days per week for 24 weeks to achieve a modest increase in mean dietary protein intake in mean dietary protein intake to a level of at least 1.3 g/kg/day. This equates to ~160 g of cooked meat per day (~45 g of protein). All participants will be asked to prepare the meals themselves and consume the meat at lunch and dinner (~80 g cooked serving per meal) as there is emerging evidence that a divided dose of protein spread throughout the day may be more effective for enhancing muscle protein synthesis than a single large dose in the evening [49]. The meat will be supplied in labelled 110 g portion packs trimmed of visible fat, and participants will select from a variety of veal, lamb or beef cuts, delivered frozen every 4 weeks by a local butcher. Participants living with a partner will also receive sufficient food supplies for their partner. For the other non-training days, participants will be advised to maintain their usual diet. All participants will be provided with instructions of how to maintain a daily protein rich diet (e.g. foods and recipes rich in protein) and will receive recipes and written dietary instructions for consuming the red meat. We have found that this dietary intervention in older women results in a modest average increase in dietary protein from 1.1 to 1.3 g/kg/day [15]. We therefore expect that participants will achieve an intake of approximately 1.3 g/kg/day for 3 days/week when exercising and around 1.1 g/kg/day for the remaining 4 days.

#### Carbohydrate control

Participants randomized to the carbohydrate control group will be instructed to consume their usual diet and consume at least one serving per day of carbohydrates (rice, potato and pasta) on each of the training days. As all adults consume some carbohydrate foods every day the provision of rice/pasta is provided to assist in keeping the participants’ dietary protein intakes towards the lower range of usual intake (<1.1 /kg/day) and to ensure that both groups receive the same level of attention. Participants in this group will be supplied with packs of pasta and rice every 3 months, and advised to consume at least one serving (~1/2 cup or ~125 ml) of cooked rice or pasta or potato on the training days (~25–35g of carbohydrates), and to focus on having larger servings of bread, cereals, rice and vegetables and smaller serves of protein foods (e.g. meat and fish). We have previously found that the provision of some rice and pasta together with this dietary advice results in no significant change in mean dietary protein intake, which remained at 1.1 g/kg/day in older women [15]. All participants will be provided with recipes and written dietary instructions for consuming a carbohydrate-rich diet and supplies for their partner where appropriate.

#### Vitamin D

All participants will be asked to take one 1000 IU vitamin D_3_ capsule (Ostelin, Australia) per day to try to ensure that serum 25-hydroxyvitamin D concentrations are ≥50 to 60 nmol/L for all participants, which is the current recommended level for Australian adults [50].

### Follow-up

At the completion of the 24-week supervised and structured intervention, all participants will be encouraged to continue to participate in the exercise program (at their own expense) and those in the red meat group will be asked to continue to consume a protein-rich diet with at least three servings of red meat per week. The primary aim of the follow-up period is to document any long-term behavioural changes with regard to maintaining participation in regular exercise and consuming a diet rich in red meat. All participants will be contacted via the telephone at weeks 36 and 48 to assess their exercise and dietary habits, using the similar methodology of 24 hr dietary recalls and a questionnaire specifically targeting the consumption of dietary protein including intake of red meat over the defined period of time since last contact.

### Outcome measures

A summary of the primary and secondary outcomes and other relevant covariates is shown in Table 1. All participants will be asked to attend Deakin University at baseline and week 24 to undertake testing, with an intermediary assessment of cognitive function conducted on-site at each health and fitness centre at week 12. With the exception of the fasted blood samples which will be collected at local pathology clinics, and the intermediary assessment, all other outcome measures will be assessed within the Centre for Physical Activity and Nutrition Research within the School of Exercise and Nutrition Sciences at Deakin University, Burwood, Melbourne, Australia.

**Table 1:** Summary of the outcome measures

Variables	Data collection method	Data collection points (weeks)				
Primary outcome measures	Baseline	Baseline	12	24	36	48
Muscle mass and cross-sectional area	Dual energy X-ray absorptiometry (DXA) total body and regional lean mass	x		x		
	Peripheral quantitative computed tomography (pQCT) scan at 25% femur site	x		x		
Muscle strength	Leg press	x		x		
	Isometric knee extensor strength	x		x		
Cognitive function	Cogstate Brief Battery computerized tests	x	x	x		
	Behaviour Rating of Inventory of Executive Function-Adult Version (BRIEF-A)	x		x		
Secondary Outcome Measures						
Muscle Function	Four-square step test	x		x		
	4 meter walk	x		x		
	30-second sit-to-stand test	x		x		
	Timed up and go test	x		x		
Fat mass and inter-/intra-muscular fat	DXA (total body and regional) and pQCT (25% femur site)	x		x		
Biochemistry and hormonal measures	Overnight fasted blood collection	x	x	x		
Neurotrophic markers	Overnight fasted blood collection	x	x	x		
Adipokines and Inflammatory markers	Overnight fasted blood collection	x	x	x		
Blood lipids	Overnight fasted blood collection	x	x	x		
Bone mineral density	DXA total body, hip and spine scan	x		x		
Blood pressure	Automated measurement	x		x		
Health related Quality of Life	Short form (SF)-36 version 2 questionnaire	x		x		
Neural plasticity and voluntary activation	Transcranial Magnetic Stimulation (TMS) *	x	x	x		
Additional Measures						
Anthropometry	Height, weight and body mass index (BMI)	x		x		
Health and Lifestyle information	Lifestyle Questionnaire	x		x		
Habitual physical activity	Community Healthy Activities Model Program for Seniors questionnaire	x		x	x	x
Memory and mood	Memory Assessment Questionnaire (MAC-Q)	x		x		
Depression and anxiety	The Hospital Anxiety and Depression Scale (HADS) questionnaire	x		x		
Diet	24-hour food diaries	Monthly			x	x
Compliance to red meat and carbohydrates	Food records	Daily calendar				
Vitamin D supplement compliance	Pill count			x		
Compliance to exercise program	Exercise cards	Monthly				
Adverse events	Exercise cards and adverse events form	Monthly				

* TMS will also be assessed at week 4 and 8

#### Primary outcome measures

The primary outcome measures will be changes in total body and regional (leg) lean tissue mass and muscle cross-sectional area, muscle strength and cognitive function.

##### Body Composition

Dual energy x-ray absorptiometry (DXA) will be used to assess total body and regional (arms and legs) lean tissue mass, fat mass and percentage body fat (Lunar Prodigy, GE Lunar Corp., Madison WI, software version 12.30.008). Muscle cross-sectional area and muscle density, as a surrogate measure of inter-/intra-muscular adiposity, at the 25 % femur site will be assessed using peripheral quantitative computed tomography (pQCT) (XCT 3000, Stratec Medizintechnik GmbH, Pforzheim, Germany). As previously reported [15], after performing a scout view of the distal end of the femur, scans will be placed at the 4 % and 25 % position of the femur. Slice thickness will be 2.3 mm, and voxel size will be set at 0.3 mm with a scanning speed of 10 mm/s. Subcutaneous fat CSA will be determined by selecting the area with thresholds –40 to +40 mg/cm^3^ HA density (contour mode 3, peel mode 1), and muscle CSA will be determined by subtracting the total bone CSA (threshold, 280 mg/cm^3^; contour mode 1, peel mode 2) and subcutaneous fat CSA from the total area of the distal femur (threshold, –40 mg/cm^3^, contour mode 3, peel mode 1). The short-term co-efficient of variation (CV) for repeated measurements of total body lean mass and fat mass in our laboratory ranges from 1.0 % to 1.7 %. The CV for femur muscle CSA is 1.3 %.

##### Muscle strength

Lower limb muscle strength will be measured from the leg press machine using a three-repetition maximum strength (3-RM) protocol. The 3-RM is the heaviest weight that an individual can complete three repetitions of an exercise whilst maintaining correct form and technique, which corresponds to ~85 % of an individual’s one repetition maximum strength (1-RM). Prior to the assessment of each individual’s 3-RM leg press strength, they will be required to complete a 5-minute warm-up on an exercise bike. To determine 3-RM, each participant will perform a warm-up set of 8-10 repetitions with a light to moderate load. After the successful completion of a further 6–8 repetitions at a heavier weight selected by the instructing researcher and following a brief rest (~1–2 minutes), the weight will be increased incrementally until only three repetitions with correct technique can be completed. To calculate 1-RM strength, the formula developed by Wathen et al. [51] will be used. In addition to the leg press, isometric knee extensor strength will be measured on the participants’ dominant leg using Lord's strap assembly incorporating a strain gauge (Neuroscience Research Australia, Sydney, Australia). Participants will have one practice trial followed by two maximal tests with a 60 second rest between each test. This test has been shown to have excellent test-retest reliability (Pearson r=0.92) [52]. For analysis, knee extension strength (kg) will be expressed per unit of lower leg length to compensate for the length of the lever arm.

##### Cognitive function

The CogState Brief Battery computerized tests (http://cogstate.com/) will be used to assess cognitive function at baseline, 12 and 24 weeks. Briefly, this is a multi-task computerised battery which provides sensitive and valid measurement of a range of different cognitive functions [53–55], including attention, processing speed, memory and executive function. It is designed to assess cognition over serial time points using a computerised system that takes 30–40 minutes to complete depending on the number of tests used. Importantly, it has been specifically designed for repeated administration over short time periods and has been validated in older adults, with minimal practice or fatigue effects, making it suited for detecting subtle change [53–55]. For this study, participants will be asked to complete five tasks from the CogState battery on a laptop with a mouse and headphones provided. Prior to each test, participants will be given a verbal set of instructions, reinforcing the instructions written on the screen. Participants will also be given a practice to ensure that they understand what is required for each test. A brief description of the five tasks is provided below.

1. The *Groton Maze Learning Task (GMLT)* is a test of executive function, memory and spatial problem solving. In this test, participants will be exposed to a 10 x 10 grid of tiles on a computer screen, with a 28-step pathway hidden among the 100 possible locations. Participants will be required to find the hidden pathway (through trial and error feedback) that extends from one corner of the grid to the other, and remember (repeat) this pathway for four subsequent trials. The task is scored using the total number of errors made by the participant on five consecutive trials at a single session. The lower the score, the better their performance.
2. The *Detection Task (DET)* is a test of reaction time measuring psychomotor function and speed of processing. In this task, participants will be shown the back of a playing card (patterned side) in the centre of the screen and asked to respond to the question “has the card turned over yet? As soon as the card is flipped to the picture side, participants will be required to press ‘yes’ on the keyboard as quickly as possible. The task is completed after 35 correct trials have been recorded. This task is scored using speed in milliseconds. The average reaction times for correct responses will be used, normalised using a log10 transformation.
3. The *Identification Task (IDN)* is a test of choice reaction time measuring visual attention. For this task, participants will be shown the back of a playing card in the centre of the screen, and asked to respond to the question ‘is the card red?” As soon as the card is flipped to the picture side, the participants have to decide as quickly as possible whether the card is red or black. If the card is red, participants will press the ‘yes’ key. If the card is black, they will press the ‘no’ key. The task will end after 30 correct responses have been recorded. The task is scored using speed in milliseconds and mean reaction times for correct responses. The average will be normalised using a log10 transformation.
4. The *One Card Learning Task (OCLT)* is a test assessing visual recognition memory and attention. In this task, participants will be shown the back of a playing card in the centre of the screen and asked to respond to the question “have you seen this card before in this task?” If the participant has seen the card, they will be required to press the ‘yes’ key, or the ‘no’ keys if they have not. In this task, six cards are randomly drawn from the deck and repeated throughout the task, and the first response will always be ‘no’, as there has been no previous card shown to the participant in the task. This task will end after 88 trials, and is scored using the proportion of correct responses, using an arcsine square-root transformation to normalise the data.
5. The *One-Back Working Memory Task (OBWMT)* is a test assessing working memory and attention. In this task participants will be shown the back of a playing card in the centre of the screen, and asked to respond to the question “is this card the same as that on the immediately previous trial?” If the answer is yes participants will be required to press the ‘yes’ key, or the ‘no’ key if the card is not the same. The first response in this task is always ‘no’ as there is no card prior to the first card. The task will end after 30 correct responses have been recorded. This test is scored using the proportion of correct responses, using an arcsine square-root transformation to normalise the data.

As previously reported [56], composite variables will be created using each individual variable within a cognitive domain. Scores will be transformed into a z-score using the mean and standard deviation (SD) of the total sample in the study and these z-scores will be averaged across tests resulting in a single composite score for processing speed/attention, memory and executive function for each participant. Higher scores on these composites indicate better performance and cognitive function.

Participants will also be required to complete a self-report of executive function at baseline and week 24 using the Behaviour Rating of Inventory of Executive Function-Adult Version (BRIEF-A). This is a self-report measure comprised of 75 questions that make up nine non-overlapping theoretically and empirically derived clinical scales that provide a measure of various aspects of executive functioning. These scales include: inhibit, self-monitor, plan/organize, shift, initiate, task monitor, emotional control, working memory, and organization of materials [57]. For each question, a response of never, sometimes or often will be recorded for how often in the last month each behaviour listed had been a problem. Each response corresponds to a number score of 1 to 4, and these are summed to provide scores for each subdomain. The inhibit, shift and emotional control subdomains are summed together to provide a behavioural regulation index (BRI) score and the other subdomains are summed to provide a metacognition index score (MI) [57]. Lastly, the BRI and MI can be summed to provide an overall global executive composite (GEC) summary score [57].

#### Secondary outcome measures

Secondary outcome measures to be assessed at baseline and week 24 (unless stated otherwise) will include: changes in fat mass, muscle function, various circulating biochemical, growth, inflammatory and neurotrophic markers, bioavailable zinc, corticospinal excitability and inhibition, blood pressure, blood lipids, memory, mood, anxiety/depression, health-related quality of life and adverse events. Other covariates and variables of interest to be assessed include: anthropometry, habitual physical activity, diet, alcohol intake and smoking status and medication use. A summary of all the outcome measures is shown in Table 1.

##### Biochemical, hormonal and inflammatory markers

All participants will be asked to attend one commercial pathology clinic with multiple collection centres where a fasted, morning (8–10am) venous blood sample will be collected at baseline, 12 and 24 weeks. All blood will be sent to a central laboratory that is a National Association of Testing Authorities Royal College of Pathologists Australasia accredited pathology laboratory. Serum total cholesterol, high-density lipoprotein (HDL)-cholesterol and triglycerides will be determined using an enzymatic colorimetric method (Roche Diagnostics, Mannheim). Low density lipoprotein (LDL)-cholesterol will be calculated by using Friedewald’s formula. High sensitivity C-reactive protein (hs-CRP) will be measured by an Immunoturbidimetric assay from Roche Diagnostics, Mannheim. Serum creatinine, urea, albumin, calcium, phosphorus, sodium, potassium and phosphorus will be analysed by using standardized techniques. A 24-hour urine sample will be collected and analysed for urinary urea, creatinine, potassium and sodium using standard techniques. Glomerular filtration rate (eGFR) will be calculated using the participants’ serum creatinine, age and gender in accordance with the appropriate Chronic Kidney Disease Epidemiology Collaboration (CKD-EPI) formula as outlined by Johnson et al [58].

Serum aliquots will also be collected and stored at –80 °C so that the following parameters can be assessed in a single batch at the completion of the study: serum IGF-1, 25-hydroxyvitamin D, homocysteine, vitamin B_12_, folate, BDNF, vascular endothelial growth factor (VEGF), adiponectin and a battery of pro-inflammatory and anti-inflammatory cytokines, including interleukin-6 (IL-6), interleukin-1 beta (IL-1β), interleukin-8 (IL-8), tumor necrosis factor-alpha (TNF-α) and interleukin-10 (IL-10). Serum bioavailable zinc will also be measured using flame atomic absorption spectrophotometry by direct aspiration (Varian SpectrAA-800).

##### Physical function

Muscle performance will be assessed using four validated tests.

The timed up-and-go test (TUG), which provides a measure of dynamic balance during three commonly performed functional activities: standing up from and sitting down in a chair, walking, and turning [59]. For this test, participants will be seated in a chair (height 45 cm) that will be placed at the end of a marked 3-metre walkway and instructed to stand up, walk at a comfortable speed for 3 meters, turn back to the chair and sit down. To minimise any ceiling effects and make the test more challenging, participants will be instructed to start counting backwards by 3’s from a random number. All participants will be given a practice trial and one test run. A stopwatch will be used to record the time taken (in seconds) to complete the test.

The 4-metre walk test will be used to assess gait speed. Participants will be required to walk in a straight line at their normal walking speed across 4 metres. Participants will begin walking 2 metres prior to and continue walking 2 metres past the 4 metre marker to ensure that a measure of usual walking speed is measured. This test will be recorded using timing gates (Swift Speedlink Performance Equipment systems). Participants will be given one practice trial before completing two test trials. The fastest time (to the nearest millisecond) will be recorded.

The four-square step test (FSST) will be used to assess dynamic standing balance and stepping speed in four different directions [60]. For this test, participants will be asked to step forward, sideways, and backwards over four canes resting flat on the floor in a cross formation as quickly as possible, moving first in a clockwise and then counter-clockwise direction to return to the starting position. After one practice trial, participants will complete two test trials and the fastest time (in seconds) taken to complete the sequence will be measured with a stopwatch and recorded as the final score.

The 30-second sit-to-stand test (STS) is a measure of lower-extremity muscle strength and function and is administered in a chair without arms [61]. Participants start from a seated position in the chair, with arms folded across the chest, and are instructed to stand fully upright and then return to the seated position at their own pace as many times as possible in 30 seconds. The final score will be the number of complete stands recorded during this time.

##### Measures of neural plasticity and voluntary activation

In a subset of approximately 30-40 participants, Transcranial Magnetic Stimulation (TMS) will be used to measure the functional properties of neurons confined to the primary motor cortex and corticospinal pathway, including corticospinal excitability, inhibition and voluntary activation. TMS is a non-invasive, painless and safe technique that is used routinely to investigate the function of the nervous system with very low risk. In this study, single and paired pulse TMS will be applied over the left motor cortex using a BiStim unit attached to two Magstim 200^2^ stimulators (Magstim, Dyfed, UK) to produce motor evoked potentials (MEPs) in the dominant (right) quadriceps. A double-cone coil will be placed over the optimal site of the left primary cortex (sites near the estimated centre of the rectus femoris - approximately 3–4 cm anterior from the vertex) to determine the optimal scalp position that produces the largest MEP amplitude. To ensure all stimuli are delivered to the optimal motor hotspot throughout testing, participants will wear a tightly fitted cap that is marked with a latitude-longitude matrix, positioned with reference to the nasion-inion and interaural lines. All TMS measures will be taken during weak muscle contraction, where participants will be instructed to exert 20 % of their pre-determined maximal voluntary torque (MVC torque), as indicated by a visual line representing voluntary knee extension force on a computer monitor connected to an isokinetic dynamometer (Biodex system 4 Pro, Biodex Medical Systems). The root mean square (rms) of the rectus femoris sEMG will be obtained prior to each TMS stimulus to ensure that there are no changes in pre-stimulus rmsEMG prior to, and following the intervention, which may artificially influence the MEP amplitude. Active motor threshold (AMT) will be determined as the minimum stimulus intensity required to elicit a MEP in the right rectus femoris of at least 200 *u*V in three of five consecutive trials [62]. The stimulus intensity will start at 50 % of maximum stimulator output (MSO) and will be altered in increments of ±1 % of MSO until the appropriate threshold level is achieved.

Single pulse input-output curves will be constructed by stimulating the motor cortex from below each participant’s AMT and by increasing the stimulator intensity by 10 % increments until MEP plateau. From the stimulus response curve, the following parameters will be determined: *MEP*_*Max*_*or peak plateau*, which provides an estimate of the proportion of motoneuron pool that was activated by TMS; *slope steepness*, which provides a measure of the neurophysiological strength of intracortical and corticospinal connections, and *half peak height slope (V*_*50*_*),* which provides a measure of the threshold characteristics of corticospinal cells and alpha motoneurons [63].

Using a paired-pulse technique, we will also measure motor cortex inhibition [short-interval intracortical inhibition (SICI) and long-interval intracortical inhibition (LICI)], which provides a measure of suppression of neuronal firing mediated by γ-amino butyric acid (GABA) receptors [64]. To establish SICI, a conditioning stimulus that is equal to 80 % of AMT will precede a supramaximal test response that will be equal to 120 % of AMT. Each pair of stimuli will be separated by a 3 millisecond interstimulus interval [65]. Similarly, to establish LICI, two supramaximal test responses set at 120 % AMT will be applied to the motor cortex, with each pair of stimuli separated by a 150 millisecond interstimulus interval [66].

Since TMS evokes a muscle twitch as a result of supramaximal stimulation, we will also measure the TMS-evoked twitch force to determine cortical voluntary activation, which provides a measure of the net motor output from the motor cortex to the muscle, as previously described [67]. To establish any changes in cortical voluntary activation, participants will be instructed to match a required force (20 %, 50 %, 75 % and 100 % maximum voluntary contraction) using a horizontal line on a computer screen as visual feedback. During each contraction, TMS will be delivered over the left hemisphere at a stimulus intensity that elicits the largest MEP to evoke superimposed twitches. Cortical voluntary activation will then be calculated using a linear regression as described by Carroll [67].

In order to identify any potential changes in the excitability of the spinal motor neuron pool, maximal electrical responses (M-waves) will be recorded by direct electrical stimulation of the femoral nerve under resting conditions. A Digitimer (Hertfordshire, UK) DS7A constant-current electrical stimulator (pulse duration 1 ms) will be used to deliver each electrical pulse. An increase in current strength will be applied to the femoral nerve until there is no further increase in the amplitude of surface electromyography (sEMG) activity response (M_MAX_) [62].

All MEPs will be recorded via sEMG activity from the right rectus femoris muscle using bipolar Ag–AgCl electrodes. These electrodes will placed on the rectus femoris, three-fifths of the distance between the anterior superior iliac spine and the upper border of the patella, with an inter-electrode distance (centre to centre) of 20 mm [65]. The reference electrode will be placed on the patella to ensure no muscle activity is recorded. All cables will be fastened with tape to prevent movement artefact. The area of electrode placement will be shaven to remove fine hair, rubbed with an abrasive rasp to remove dead skin, and then cleaned with 70 % isopropyl alcohol. sEMG signals will be amplified (1000×), bandpass filtered (high pass at 13 Hz, low pass at 1000 Hz), digitized online at 2 kHz, recorded and analyzed using PowerLab 4/35.

##### Blood pressure

Systolic and diastolic blood pressure will be measured using an automated blood pressure monitor (A&D Instruments, Oxon, UK) after a 5-minute rest period seated in a quiet room. A total of four measurements will be taken with a 2-minute interval between readings; the mean of the final three readings will be used in the analysis.

##### Health-related quality of life

Health-related quality of life (QoL) will be assessed using the Short-Form (SF)-36 (version 2) questionnaire, with permission from the Medical Outcomes Trust (Boston, MA, USA), and reported using Australian norm-based scores according to previously published guidelines [68, 69]. From the 36 items, scores will be calculated for the following eight domains: physical functioning, role-physical, bodily pain, general health, vitality, social functioning, role-emotional and mental health [69]. The data will be normalised to reported data from the 2004 South Australian Health Omnibus survey to allow for appropriate and meaningful comparisons for changes in scores across the eight domains and summary scores [68]. This will allow for the changes in domains to be assessed meaningfully and will account for cultural differences in various populations in regard to the meaning of QoL. Summary measures of the physical and mental components of the survey will be calculated based on a factor analysis of the eight domains among participants in the 2004 South Australian Health Omnibus Survey, resulting in two separate overall summary scores: the physical component summary (PCS) and the mental component summary (MCS) scores [68]. The use of norm-based weights gives each domain score a mean of 50 and a standard deviation (SD) of 10, allowing change in scores to be assessed on a comparable scale.

#### Additional measures

##### Memory, depression and anxiety

The Memory Assessment Questionnaire (MAC-Q) [70] will be used as a measure of subjective memory complaint severity. This tool comprises six questions related to memory functioning in everyday situations in which the participants are asked to compare and rate their current ability compared to when they were aged 40. The total score on the MAC-Q ranges from 7 to 35, where greater scores indicate subjective memory loss. Scores greater than or equal to 25 have been found to be suggestive of age-associated memory impairment.

The Hospital Anxiety and Depression Scale (HADS) questionnaire will be used to measure psychological functioning (anxiety and depression) [71]. The HADS questionnaire is a self-report rating scale consisting of 14 items on a four-point likert scale (range: 0-3 where a higher score represents more severe depression or anxiety), which includes seven questions evaluating cognitive and emotional aspects of anxiety, and seven questions assessing the cognitive and emotional aspects of depression. The results will be reported as the sum of all 14 items, and the sum for each of the two subscales of seven items.

##### Dietary habits

Dietary habits will be assessed from two 24-hour dietary diaries completed by the participants on two consecutive days at baseline and at weeks 4, 8, 12, 16, 20 and 24 and then again at weeks 36 and 48. Participants in the lean red meat group will complete one 24-hour food diary on a day that they consume their red meat and the other 24-hour food diary on a day that they do not consume red meat as part of the intervention. Participants will be required to provide as much detail as possible regarding brand names, cooking methods and recipes for foods and beverages consumed in the past 24 hours. Household measures including measuring cups, plates, bowls and glasses will be used to assist in estimating food portion sizes. Information from these diet history diaries will be checked by the research staff and used to assess total energy and nutrient intakes using Foodworks 7 (Foodworks, Xyris Software, Highgate Hill, Australia).

##### Anthropometry

Height will be measured to the nearest 0.1 cm with a wall-mounted stadiometer and bodyweight to the nearest 0.1 kg using calibrated electronic digital scales.

##### Physical activity

The Community Healthy Activities Model Program for Seniors (CHAMPS) physical activity questionnaire will be used to assess total leisure and recreational physical activity time. This questionnaire has been specifically designed for use in older adults and found to be reliable, valid and sensitive to change [72]. Participants will document their weekly frequency and duration of participation in a ‘typical week’ of the preceding four weeks. The results will be reported as hours per week and, estimated kilojoules per week spent in moderate to high intensity activities.

##### Health and medical history and medication use

A lifestyle questionnaire will be used to obtain information on education background, history of disease(s)/illnesses, falls and fractures, smoking history, current use of medication and dietary supplement use, average weekly alcohol consumption, and weekly television viewing and sitting time, and sun exposure habits. Information on any alterations to, or new, medication prescribed by the participants’ doctor during the study will also be collected by research staff via the monthly phone calls. Information including medication name, dose prescribed, and daily quantity taken will be recorded.

##### Compliance

Exercise cards completed by the participants and monitored by the exercise trainers will be used to monitor adherence to the exercise program. Moreover, at each training session the exercise trainers will document attendance/non-attendance for each participant, and will contact the research staff if participants miss multiple sessions consecutively. The exercise cards will be collected by the research staff from each health and fitness centre at approximately monthly intervals throughout the intervention for review. To monitor the consumption of lean red meat, participants will be asked to record all meat products consumed (from that supplied by the investigators) on a compliance calendar, which will be collected every month. Participants in the carbohydrate control group will also be asked to complete a checklist of carbohydrate-rich meals (pasta and rice) on a daily calendar. Compliance with the vitamin D supplements will be checked by counting returned tablets at after 6 months.

##### Adverse events

At the end of each 4-week period, participants will be required to document any exercise-related adverse events including any injuries related to the exercise program. An adverse event will be defined as an intervention-related event resulting in absence from, or modification to, the exercise program. All exercise-related adverse events forms will be collected monthly so that the researchers can follow-up on any adverse events. Diet-related adverse events (intervention-related events resulting in modification of dietary instructions) will be collected via a telephone administered questionnaire every 8-weeks.

### Sample size calculations

The number of participants required for this study is based on power calculations for the expected changes in total body lean mass, lower limb muscle strength and cognitive function, particularly executive function. Based on our previous study in elderly women [15], we estimate that 152 older adults in each group will provide >80 % power (P<0.05, two tailed) to detect a 0.5 kg and 18 % differences for the change in total body lean mass and leg muscle strength between the red meat and control group, assuming a standard deviation of 0.9 kg and 30 %, respectively. This calculation allows for a possible 20 % drop-out or loss to follow-up. For measures of cognitive function, it was difficult to provide an accurate estimate of the magnitude of any intervention effect (effect size) because this study is the first to examine the effects of exercise combined with increased dietary protein compared to exercise alone in older adults. Previous research examining the effects of exercise on measures of cognitive function report moderate effect sizes (*d* = 0.41 to 0.59) following PRT or aerobic training alone or in combination [25, 26, 73], particularly on measures of executive function and processing speed. Whether increased dietary protein can improve cognitive function in older adults remains uncertain, but we hypothesize that exercise combined with a modest increase in dietary protein (1.3 g/kg/day) will lead to a medium effect. Using data from a pilot study in 72 healthy older adults aged 65 years and over in which the mean (standard deviation, SD) scores on the CogState Groton Maze Learning Task (a measure of executive function) and the Detection Task (a measure of reaction time and processing speed) were 64 (18) and 2.56 (0.07) respectively, we anticipate that a sample size of at least 140 will provide approximately 80 % power (P<0.05, two-tailed) to determine a medium effect size (*f* = 0.20) assuming a 0.6 correlation among repeated measures.

### Statistical analyses

The primary statistical analysis will be conducted on an intention-to-treat basis using STATA statistical software release 13.0 (STATA, College Station, TX, USA). For participants with at least 66 % compliance to the exercise program (two out of three sessions per week) and 80 % compliance with the lean red meat, per protocol analyses will also be performed. Descriptive statistics will be used to compare the background variables and baseline measures between the groups, and any imbalances will be adjusted for during subsequent analyses. Wherever possible, we will obtain endpoint measures from all withdrawals and include all randomised participants in our final data analysis. Any missing data for participants that are lost to follow-up will be handled with multiple imputation, but sensitivity analysis will be conducted to evaluate the effect of potential non-random attrition. All data will be checked for normality prior to analysis, and data that is skewed will be log transformed prior to analysis. Baseline measures and changes in outcome variables will be presented as means ± SD or 95 % confidence intervals. The effects of the intervention will be assessed using linear mixed models with random effects. Subsequent adjusted analysis for potential covariates (age, sex, race/ethnicity, cardiovascular disease risk, depression/anxiety, changes in medication and change in habitual physical activity or diet) will also be conducted. Multiple regression analysis will be used to investigate whether body composition, circulating neurotrophic, growth or inflammatory markers, bioavailable zinc and cardiovascular risk factors are associated with changes in cognitive function. The significance level will be set at P<0.05 or smaller if adjustments are made for multiple comparisons.

## Discussion

This community-based RCT will be the first to evaluate whether a multi-modal exercise program incorporating PRT in combination with increased dietary protein achieved through the consumption of two ~80 g servings of cooked lean red meat on each of the three training days is a safe and effective strategy to enhance muscle mass, size and strength and cognitive function in community-dwelling older adults. In addition, this study will determine whether such an intervention translates into long-term behavioural changes with regard to adopting a healthier lifestyle that incorporates regular participation in PRT and consumption of a diet high in protein (red meat). This is important because age-related muscle wasting and a decline in cognitive function have both been implicated in the development and progression of many chronic diseases, including falls and dementia. From a public health perspective, if the intervention proves to be effective, the pragmatic nature of the intervention, with the exercise conducted within local community-based health and fitness centres and dietary protein increased through the use of lean red meat at an intake equivalent to the current Australian dietary guidelines of 3–4 serves per week, should ensure that the program can be easily implemented and adopted and is applicable in real life.

Another strength of the study is that our secondary outcomes will provide a unique opportunity to: 1) evaluate the effects of the intervention on neural plasticity, and 2) identify potential biological determinants of exercise and protein-induced changes in cognitive function (and skeletal muscle health) in older adults. Specifically, this study will be one of the first to determine whether combining exercise with increased dietary protein (lean red meat) can have a positive effect on a range of neurobiological markers, including various circulating growth and neurotrophic factors (e.g., IGF-1, BDNF, VEGF) and systemic inflammatory cytokines in older adults, and whether any changes in these biological determinants in response to the intervention are associated with any changes in cognitive function and neural plasticity.

In summary, we believe that the findings from this study will form the basis for more targeted nutrition and exercise guidelines for the management and prevention of age-related changes in muscle and neural health as well as cognitive function in older people. We anticipate that the findings from this study will provide direct evidence that the current guidelines to consume no more than 455 g of lean red meat is a safe, acceptable and effective dose and source of protein that can have beneficial effects on improving multiple health outcomes when combined with regular exercise. In addition, we expect that the results will also show that there are no adverse effect of this approach on cardiovascular-related health outcomes or kidney function. This is important because there are some concerns that a high red meat diet is associated with a number of adverse health outcomes, including an increased risk of heart disease, type 2 diabetes and certain cancers.

## Trial status

Recruitment is currently underway and a number of participants have commenced the study.

## Abbreviations

PRT: Progressive resistance training
IGF-1: Insulin-like growth factor-1
BDNF: Brain-derived growth factor
CRP: C-reactive protein
IL-6: Interleukin-6
TNF-α: Tumor necrosis factor-alpha
ADL: Activities of daily living
MPS: Muscle protein synthesis
MPB: Muscle protein breakdown
RCT: Randomized controlled trial
TMS: Transcranial magnetic stimulation
RT: Resistance training
CRT: Control resistance training
SPMSQ: Short portable mental status questionnaire
GDS: Geriatric depression scale
ACSM: American College of Sports Medicine
eGFR: Estimated glomerular filtration rate
RPE: Rating of perceived exertion
DXA: Dual energy X-ray absorptiometry
pQCT: Peripheral quantitative computed tomography
CSA: Cross-sectional area
HA: Hydroxyapatite equivalent densities
CV: Co-efficient of variation
3-RM: Three repetition maximum
BRIEF-A: Behaviour Rating of Inventory of Executive Function-Adult Version
BRI: Behavioural regulation index
MI: Metacognition index
GEC: Global executive composite score
LDL: Low density lipoprotein
HDL: High density lipoprotein
VEGF: Vascular endothelial growth factor
IL-1β: Interleukin-1 beta
IL-8: Interleukin-8
IL-10: Interleukin-10
STS: Sit-to-stand
FSST: Four-square step test
TUG: Timed-up-and-go
MEP: Motor evoked potential
AMT: Active motor threshold
rms: Root mean square
SICI: Short-interval intracortical inhibition
LICI: Long-interval intracortical inhibition
MVC: Maximum voluntary torque
sEMG: Surface electromyography
QoL: Quality of life
SF-36 v2: Short-form 36 version 2
PCA: Physical component summary
MCS: Mental component summary
MAC-Q: Memory assessment questionnaire
HADS: Hospital Anxiety and Depression Scale
CHAMPS: Community Healthy Activities Model Program for Seniors
